# Navigating the Uncharted: Rethinking Cardioprotective Therapy Discontinuation in Cancer Therapeutics–Related Cardiac Dysfunction

**DOI:** 10.1016/j.jaccao.2024.11.009

**Published:** 2025-02-04

**Authors:** Beina Hui, Weibin Hu, Yongkai Lu

The study by Park et al^1^ offers valuable insights into the prognosis of patients with improved cancer therapeutics–related cardiac dysfunction (CTRCD) following cardioprotective therapy (CPT) withdrawal. However, the complexity of CTRCD demands a more nuanced approach to CPT cessation, as the study’s findings on left ventricular ejection fraction (LVEF) decline among patients with baseline LVEF <45% highlight the risks of premature discontinuation. This underscores the need for individualized protocols in subsequent cardio-oncology guidelines.^1^

A limitation of the study is its heterogeneous patient cohort, encompassing multiple cancer types and treatments with varying cardiotoxic potential. This variability may obscure the influence of specific therapies on LVEF outcomes post-CPT withdrawal. Future research in larger cohorts could stratify patients by cancer type and therapeutic regimen, including HER2-targeted therapies and anthracycline-based protocols, to identify subgroup-specific risks.^2^^,^^3^ Such an approach could refine the criteria for safe CPT discontinuation, potentially reducing adverse events in high-risk patients.

Moreover, the lack of cardiac biomarkers, such as N-terminal pro–B-type natriuretic peptide (NT-proBNP), in this study is a missed opportunity to explore markers predictive of relapse. Given the role of NT-proBNP in heart failure monitoring, incorporating such biomarkers could enable real-time adjustments to CPT, enhancing patient safety during withdrawal.^4^

Finally, although the investigators emphasize the economic and clinical burden of long-term CPT, additional research on the long-term outcomes of staggered or partial CPT reduction protocols could provide alternatives for patients who may not tolerate full discontinuation. This strategy could help balance patient quality of life with cardiovascular health, an essential consideration as cancer survival rates continue to improve.
